# Supplementary material for: Liraglutide Suppresses Tau Hyperphosphorylation, Amyloid Beta Accumulation through Regulating Neuronal Insulin Signaling and BACE-1 Activity
Source: Int J Mol Sci. 2020 Mar 3;21(5):1725. doi: 10.3390/ijms21051725 (PMC7084306; doi:10.3390/ijms21051725)
Supplement: Supplementary file 1 [file ijms-21-01725-s001.pdf]

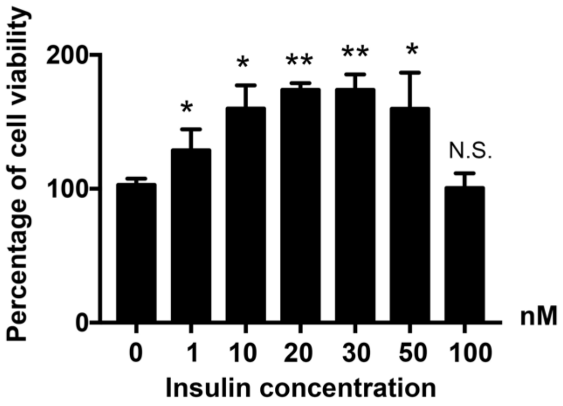

Figure S1. In vitro cell cytotoxicity assay of SH-SY5Y cells with 48-h insulin induction. Insulin at 1, 10, 20, 30, 50, and 100 nM was applied onto SH-SY5Y cells for two days. Cell viability was determined by measuring the percentage of cell viability compared to control (0 nM insulin). Values are mean ± SEM * $p < 0.05$, ** $p < 0.01$. All experiments shown were from $n = 3$.

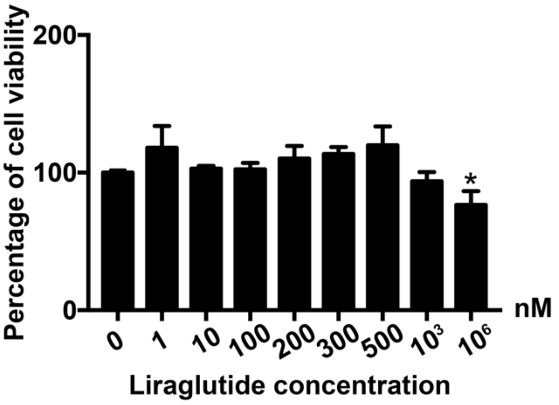

Figure S2. In vitro cell cytotoxicity assay of SH-SY5Y cells on 24 h liraglutide treatment. Liraglutide at 1, 10, 100, 200, 300, 500, 10^3, and 10^6 nM was applied onto SH-SY5Y cells for two days. Cell viability was determined by measuring the percentage of cell viability compared to control (0 nM liraglutide). Values are mean ± SEM * $p < 0.05$. All experiments shown were from $n = 3$.
